# Macromolecular shape and interactions in layer-by-layer assemblies within cylindrical nanopores

**DOI:** 10.3762/bjnano.3.54

**Published:** 2012-06-28

**Authors:** Thomas D Lazzara, K H Aaron Lau, Wolfgang Knoll, Andreas Janshoff, Claudia Steinem

**Affiliations:** 1 Institute of Organic and Biomolecular Chemistry, Tammannstr. 2, 37077 Göttingen, Germany; 2 Department of Biomedical Engineering, Northwestern University, 2145 Sheridan Road, Evanston, IL 60202, USA; 3 Austrian Institute of Technology, Donau City Str. 1, 1220 Vienna, Austria,; 4 Institute of Physical Chemistry, Tammannstr. 6, 37077 Göttingen, Germany

**Keywords:** avidin-biotin, dendrimers, nanoporous substrates, optical lightmode waveguide spectroscopy, polyelectrolytes

## Abstract

Layer-by-layer (LbL) deposition of polyelectrolytes and proteins within the cylindrical nanopores of anodic aluminum oxide (AAO) membranes was studied by optical waveguide spectroscopy (OWS). AAO has aligned cylindrical, nonintersecting pores with a defined pore diameter *d*_0_ and functions as a planar optical waveguide so as to monitor, in situ, the LbL process by OWS. The LbL deposition of globular proteins, i.e., avidin and biotinylated bovine serum albumin was compared with that of linear polyelectrolytes (linear-PEs), both species being of similar molecular weight. LbL deposition within the cylindrical AAO geometry for different pore diameters (*d*_0_ = 25–80 nm) for the various macromolecular species, showed that the multilayer film growth was inhibited at different maximum numbers of LbL steps (*n*_max_). The value of *n*_max_ was greatest for linear-PEs, while proteins had a lower value. The cylindrical pore geometry imposes a physical limit to LbL growth such that *n*_max_ is strongly dependent on the overall internal structure of the LbL film. For all macromolecular species, deposition was inhibited in native AAO, having pores of *d*_0_ = 25–30 nm. Both, OWS and scanning electron microscopy showed that LbL growth in larger AAO pores (*d*_0_ > 25–30 nm) became inhibited when approaching a pore diameter of *d*_eff,n_max_ = 25–35 nm, a similar size to that of native AAO pores, with *d*_0_ = 25–30 nm. For a reasonable estimation of *d*_eff,n_max_, the actual volume occupied by a macromolecular assembly must be taken into consideration. The results clearly show that electrostatic LbL allowed for compact macromolecular layers, whereas proteins formed loosely packed multilayers.

## Introduction

Layer-by-layer (LbL) deposition is a versatile technique [1–2] to create functional submicrometer thin films and consists of the sequential deposition of functional adsorbing components, to generate multilayered structures. Different functional materials can be stepwise incorporated by LbL, within a single surface structure by electrostatic self-assembly [3–4], molecular-recognition pairs [5–7], or covalent-bond formation [8]. Homogeneous and heterogeneous layered mixing of nanometer-sized species, such as polyelectrolytes, proteins and nanoparticles, has led to various technologically relevant surface coatings [9–13], to the preparation of capsules [14–15] and to functional one-dimensional materials, such as nanotubes, by template replication [16–18].

LbL structures on flat surfaces can be well characterized with subnanometer sensitivity by using a number of surface analysis techniques, such as surface plasmon resonance, atomic force microscopy or ellipsometry [19–20]. For LbL structures formed inside porous systems, such as within films of colloidal particles or cylindrical nanoporous membranes, the direct investigation of surface processes occurring within nanosized pores has been hampered by the limited availability of in situ, high-sensitivity, surface characterization techniques to monitor changes occurring inside the porous morphologies. Techniques such as optical waveguide spectroscopy [21–24] (OWS) and thin-film reflectometry [25–26] have recently been used to independently characterize the thickness and refractive index of optically transparent dielectric thin films.

Here, we studied the formation of LbL assemblies, obtained by the sequential adsorption of macromolecules within the nanopores of porous anodic aluminum oxide (AAO). The shape and the nature of the interactions between macromolecules were varied. AAO is widely used due to its self-organized, predictable structure, which is composed of nonintersecting, hexagonally close-packed, cylindrical pores running straight through the AAO membrane thickness, with conveniently adjustable monodisperse pore diameters, degree of lattice spacing, and membrane thickness [27–29], making it well suited as a model nanoporous system [30–32].

LbL of polyelectrolyte species within AAO has been experimentally shown to be strongly influenced by pH [16], ionic strength [23], and steric limitations [23], such that interior deposition can be partially or completely inhibited. On the one hand, electrostatics play a pivotal role in the multilayer growth process involving charged species, in which polyelectrolyte strength, polyelectrolyte chemical structure and solution ionic strength can strongly influence deposition within the nanopores [32]. On the other hand, the confined cylindrical nanopore environment imposes a steric constraint, in which pore walls are physical barriers that limit the amount of material that can be stepwise incorporated. Confinement in nanoporous environments can, for example, decrease the apparent p*K*_a_ values of cationic polymer brushes, a priori polymerized in pores with 10–40 nm pore diameters, by more than a full pH unit [33]. Dobrynin and co-workers have recently simulated the pore-filling behavior during LbL deposition of both nanoparticle–polyelectrolyte [34] and polyelectrolyte–polyelectrolyte structures [35]. For the fabrication of LbL structures, both steric and electrostatic considerations related to the confined nanoporous geometry generally determine at which point the deposition becomes hindered.

We show here that at the molecular level, parameters such as the macromolecular structure and shape of the LbL building block, as well as the nature of the self-assembly interactions are factors that influence the geometrical arrangement and shape of the growing multilayer film, and therefore modify the point at which hindrance to pore-filling is reached. The LbL deposition of linear polyelectrolytes (linear-PEs) and of globular proteins within AAO nanopores was contrasted to the previously reported behavior of dendrimer polyelectrolytes (dendrimer-PEs) [23]. Deposition of these macromolecules in AAO with pore diameters of *d*_0_ = 63–66 nm, was initially compared with deposition on a planar, charged gold surface. LbL experiments were then carried out in pores with different diameters *d*_0_, ranging from 25 to 80 nm, until the interior deposition became inhibited. For the cylindrical pore geometry of AAO, the interior deposition was hindered for pore diameters below 30 nm, regardless of the macromolecular structure. In addition, ex situ scanning electron microscopy (SEM) was employed to corroborate the in situ OWS results for linear-PEs.

## Results and Discussion

**Scheme 1:**
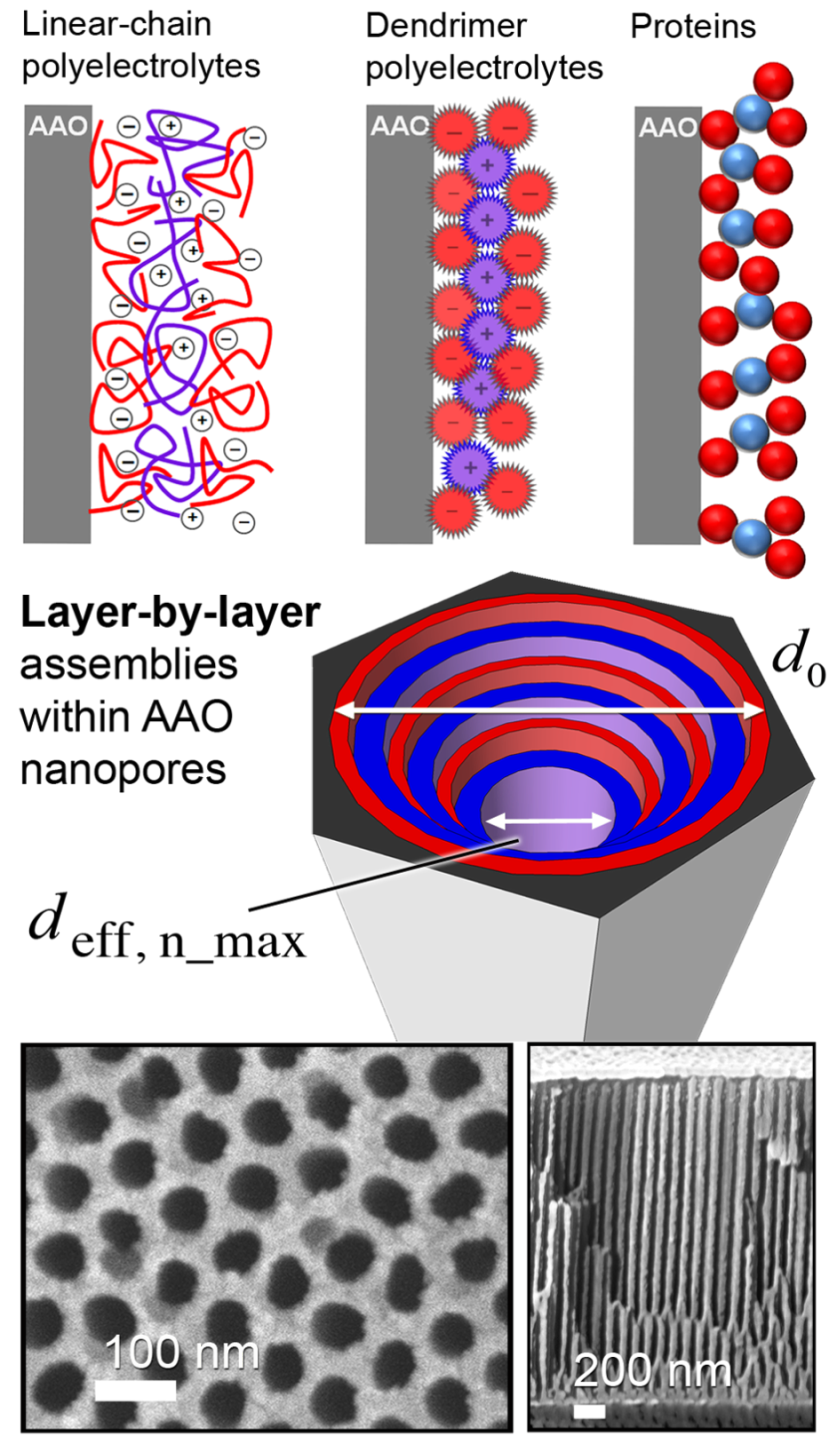
(Top) Schematic of the layer-by-layer (LbL) structure for different types of macromolecules deposited on the interior AAO pore walls. Polyelectrolyte species (linear chains and dendrimers) adsorb electrostatically, while globular proteins bind by molecular recognition. The LbL process was performed until no further material could be incorporated within the AAO, reaching *d*_eff,n_max_. (Bottom) SEM of AAO used, shown from the top (left) and in cross-section (right).

For our studies on LbL deposition of globular proteins and linear-PE multilayers, we used the nanopores of anodic aluminum oxide (AAO). Scheme 1 (top) shows the general expected internal structure after LbL deposition in AAO nanopores. A two-step anodization process of the AAO ensured highly ordered pores with a low pore-diameter (*d*_0_) size distribution (Scheme 1, bottom). The resulting AAO substrates had an interpore distance of *p* = 95–105 nm and a thickness of *h =* 3.2–3.8 µm, while the pore diameters were tuned between *d*_0_ = 25–80 nm by isotropic pore-widening in phosphoric acid. Before pore diameter adjustment, they were covered with a thin metal coupling layer on the aluminum oxide barrier side (bottom) and then mounted on glass supports by using an optical adhesive [34]. This allowed the characterization of the AAO refractive index and the in situ monitoring of the macromolecule adsorption kinetics by optical waveguide spectroscopy (OWS). Nanoporosity ensures minimal scattering losses at visible or longer wavelengths, and the Maxwell–Garnett effective medium theory (EMT) can be used to estimate the amount of macromolecular material adsorbed within the AAO nanopores from the experimentally observed changes in the refractive index [22]. This EMT approach relies on the volume fraction contribution of an adlayer on the pore walls, representing an average overall increase in the refractive index of the entire porous material. The approximation is based on the assumption that contiguous layers of uniform thickness are deposited. The film thickness (*t*_optical_) obtained by the optical measurements was estimated assuming that a uniform deposition along the entire length of the pore was achieved. For the porous AAO, *t*_optical_ was obtained by fitting the Maxwell–Garnett EMT to the experimentally observed changes of the dielectric constant, providing an average adlayer thickness on the inner pore walls (see Experimental) [22–23].

### Layer-by-layer growth

The influence of the geometric confinement on the LbL process was elucidated by comparing the deposition of different macromolecular species on flat surfaces with that in nanopores. LbL deposition on flat surfaces was measured by surface plasmon resonance (SPR) by using gold substrates with a negatively charged self-assembled monolayer of mercaptohexadecanoic acid. The formation of protein multilayers was achieved by using molecular recognition of biotinylated-bovine serum albumin (b-BSA) by avidin. Avidin has four biotin-binding sites, whereas the b-BSA used has 13 biotin molecules per protein on average. Avidin with a mass of *M*_W_ = 66–69 kDa, and which is positively charged at neutral pH, was first adsorbed onto the negatively charged surface, followed by b-BSA (*M*_W_ = 67 kDa) adsorption through molecular recognition. Linear-polyelectrolytes (linear-PEs) self-assembled into multilayers by electrostatic interactions between 70 kDa poly(sodium 4-styrene sulfonate) (PSS) and 50–65 kDa poly(allyl amine) hydrochloride (PAH). The positively charged macromolecules were deposited first on the self-assembled mercaptohexadecanoic acid monolayer on gold, followed by the negatively charged linear-PEs. For the porous AAO samples, protein multilayers were grown by first adsorbing avidin electrostatically on the untreated AAO surface, which is negatively charged. For the polyelectrolyte species, the macromolecules were deposited on a positively charged AAO surface obtained by silanization with (3-aminopropyl)-triethoxysilane. In all LbL steps, each adsorption step was continued until the adsorption kinetics showed that saturation was reached. The ionic strength was kept sufficiently high to screen the electrostatic repulsion between same-charge molecules to achieve optimal pore-loading conditions.

**Figure 1:**
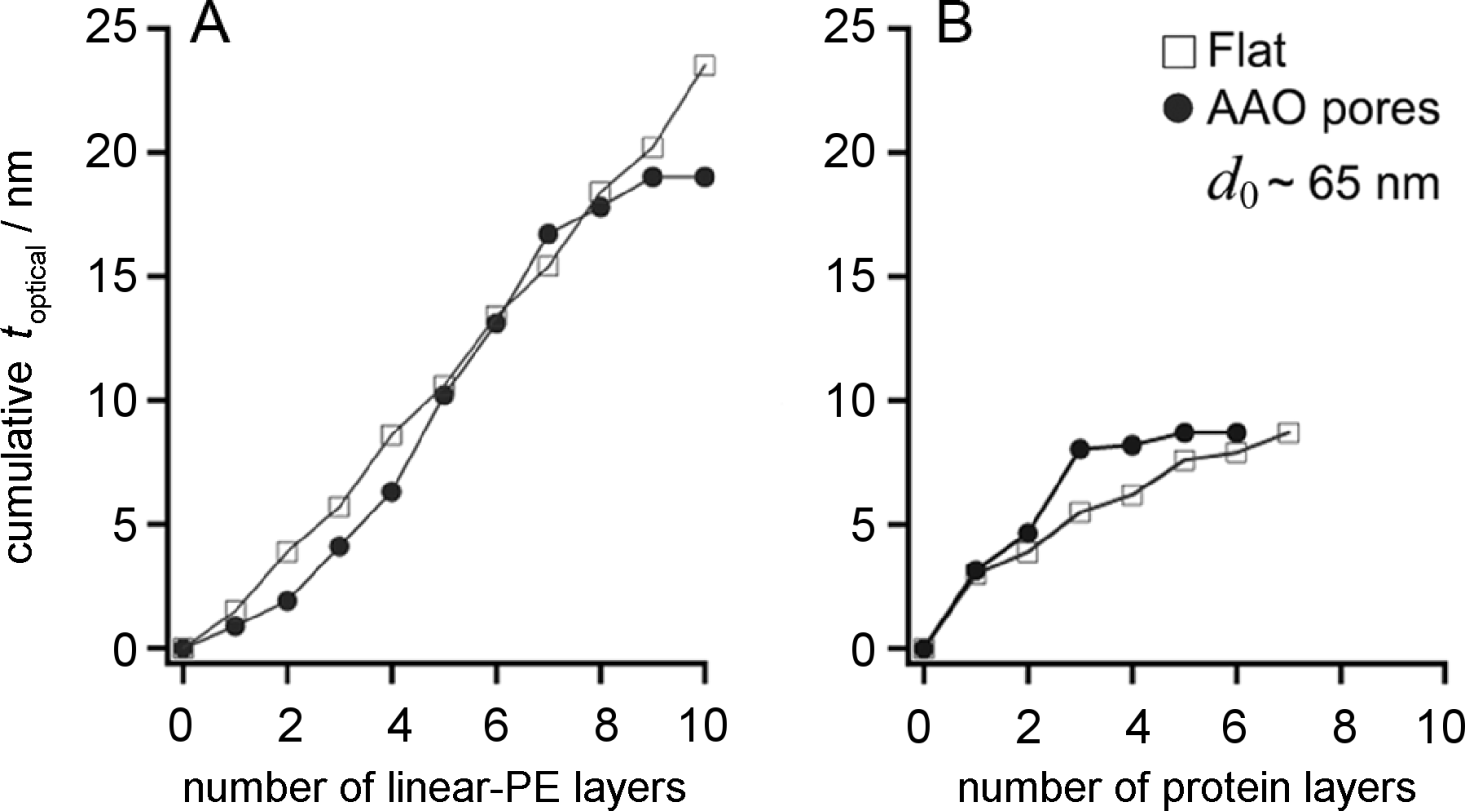
Cumulative optical LbL film thickness (*t*_optical_) for a flat and porous (AAO with *d*_0_ = 65 nm) substrate, for (A) linear-PEs and (B) proteins. LbL growth becomes hindered in cylindrical nanopores after a certain number of deposited layers.

In Figure 1, the cumulative optical film thickness *t*_optical_ obtained for the LbL growth of macromolecules, on a flat surface and within AAO nanopores of 65 nm diameter, is shown as a function of the number of added layers for both, linear-PEs (ε_linear-PEs_ = 2.15) [36] and proteins (ε_proteins_ = 2.10) [22]. The estimation of *t*_optical_ was made using the same value of dielectric constant for each of the LbL species, in both the planar- and the porous-surface estimates. Comparing the LbL growth on a flat surface versus that within nanopores clearly illustrates how the cylindrical AAO pore geometry imposes a steric limit that terminates the growth of the LbL film after a certain maximum number of deposition steps (*n*_max_), unlike deposition on a flat surface, which has in principle no steric limit to the number of possible deposition steps. Although the macromolecules discussed here were approximately the same globular size in solution, different *n*_max_ were observed for similar pore diameters *d*_0_ = 65 nm. For linear-PEs *n*_max_ = 9, whereas this value was significantly lower for globular proteins (*n*_max_ = 3).

**Figure 2:**
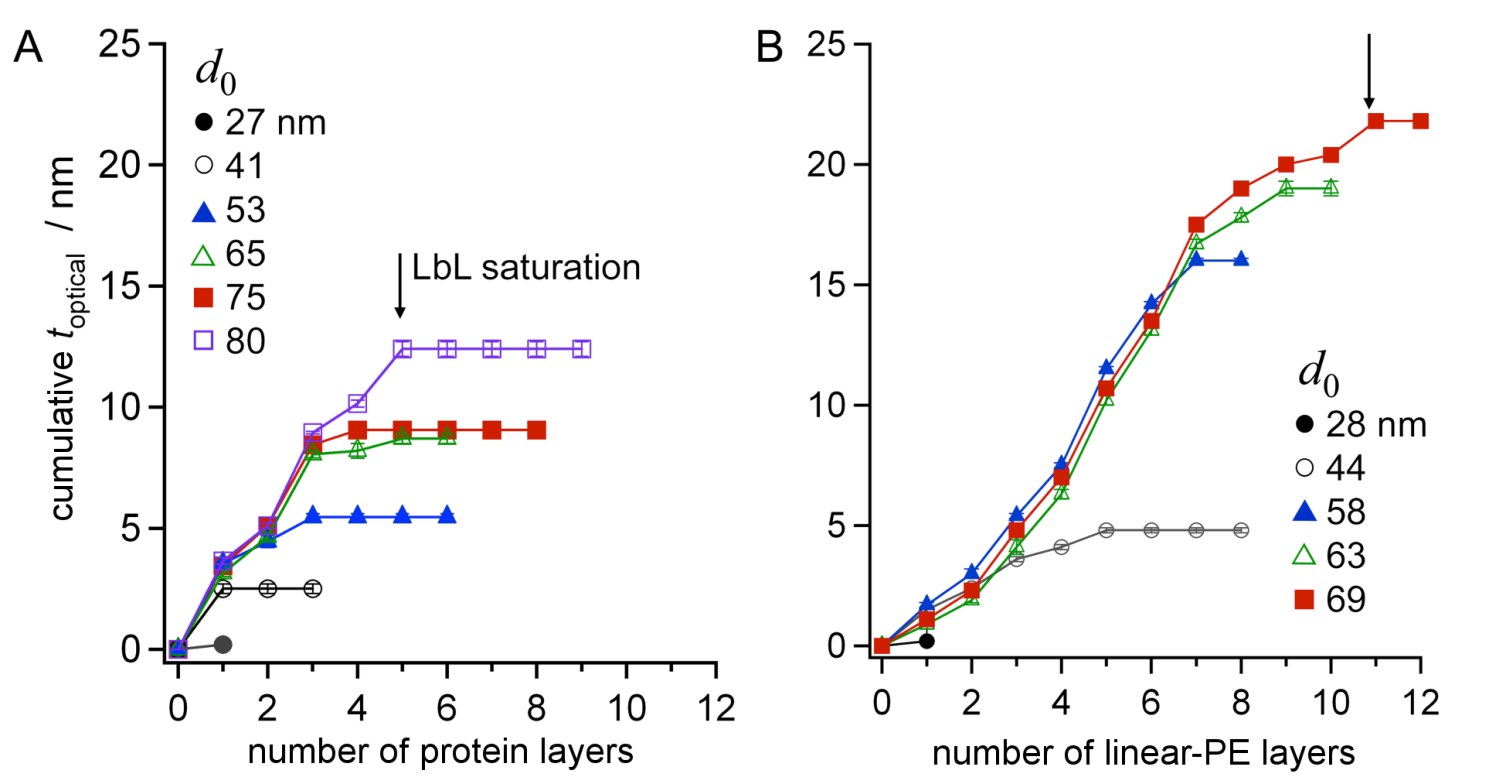
Cumulative optical thickness *t*_optical_ of the LbL multilayer film on the inner surface of the AAO cylindrical nanopores as a function of the number of deposition steps and the AAO pore diameter, for (A) proteins and (B) linear polyelectrolytes.

Interestingly, for LbL deposition of dendrimer-polyelectrolytes (Scheme 1) in AAO with pores of the same size, an *n*_max_ = 7 was found (Supporting Information File 1, Figure S1) [23]. These polyelectrolyte dendrimers were N,N-disubstituted hydrazine phosphorus-containing dendrimers of the fourth generation (G4) [37]. Each dendrimer had 96 peripheral charged groups, which were either all cationic or all anionic in nature (G4(+) = G_4_(NH^+^Et_2_Cl^−^)_96_, *M*_w_ = 32.3 kDa; G4(−) = G_4_(CHCOO^−^Na^+^)_96_, *M*_w_ = 36 kDa). The mass of these molecules is only half of that of the proteins and linear-PEs, respectively, which would imply that more layers could be deposited in the AAO pores. However, the smaller *n*_max_ compared to that obtained for the linear PEs suggests that their structure in the adsorbed state is more globular. This influence of the shape of the adsorbed molecules on *n*_max_ was even more clearly observed when contrasting the LbL growth of proteins and linear-PEs over a range of pore diameters *d*_0_ = 25–80 nm. The cumulative increase in *t*_optical_ as a function of the number of added macromolecular layers is shown in Figure 2 for proteins and linear-PEs.

The value of *n*_max_ increases with larger values of *d*_0_ for both macromolecules. The striking difference between these two macromolecules is that saturation occurs at significantly lower *n*_max_ values for proteins than for linear-PEs, at similar *d*_0_. For *d*_0_ = 80 nm, only 5 protein LbL layers could be grown, whereas 11 layers of PSS and PAH were deposited within *d*_0_ = 69 nm pores.

The overall multilayer growth process was different for the two molecular species shown in Figure 2. The deposition process of proteins and linear-PEs in cylindrical pores was first characterized by a linear behavior, similar to that for a flat surface (Figure 1). Some deviations were observed for the initial deposition steps for the linear-PEs due to differences in the initial surface charge density, i.e., the number of positively charged silanes on alumina versus negatively charged thiols on gold [38]. Then, for protein multilayers, the LbL deposition saturates rather quickly indicated by *t*_optical_, which does not change upon further addition of protein (Figure 2). For linear-PEs, a transition period proceeds for a few deposition steps, characterized by a reduction in *t*_optical_ per deposited layer. This reduced deposition is likely due to the onset of hindered diffusion within the nanopore near the pore entrance, which decreases the total amount of material being adsorbed within the porous matrix. Finally, saturation is reached at *n*_max_, at which point electrostatic repulsion between same-charge species inhibits the deposition of additional material within the nanopores. The observed behavior, in which the LbL growth in the cylindrical nanopores only proceeded for a certain *n*_max_ and terminated before the pore was completely occluded, was also observed for dendrimer-PEs (Supporting Information File 1, Figure S2). Similar experiments involving the formation of polymer nanotubes by LbL of poly(acrylic acid) and PAH, similarly showed that LbL terminates before the pores become completely occluded [16].

**Figure 3:**
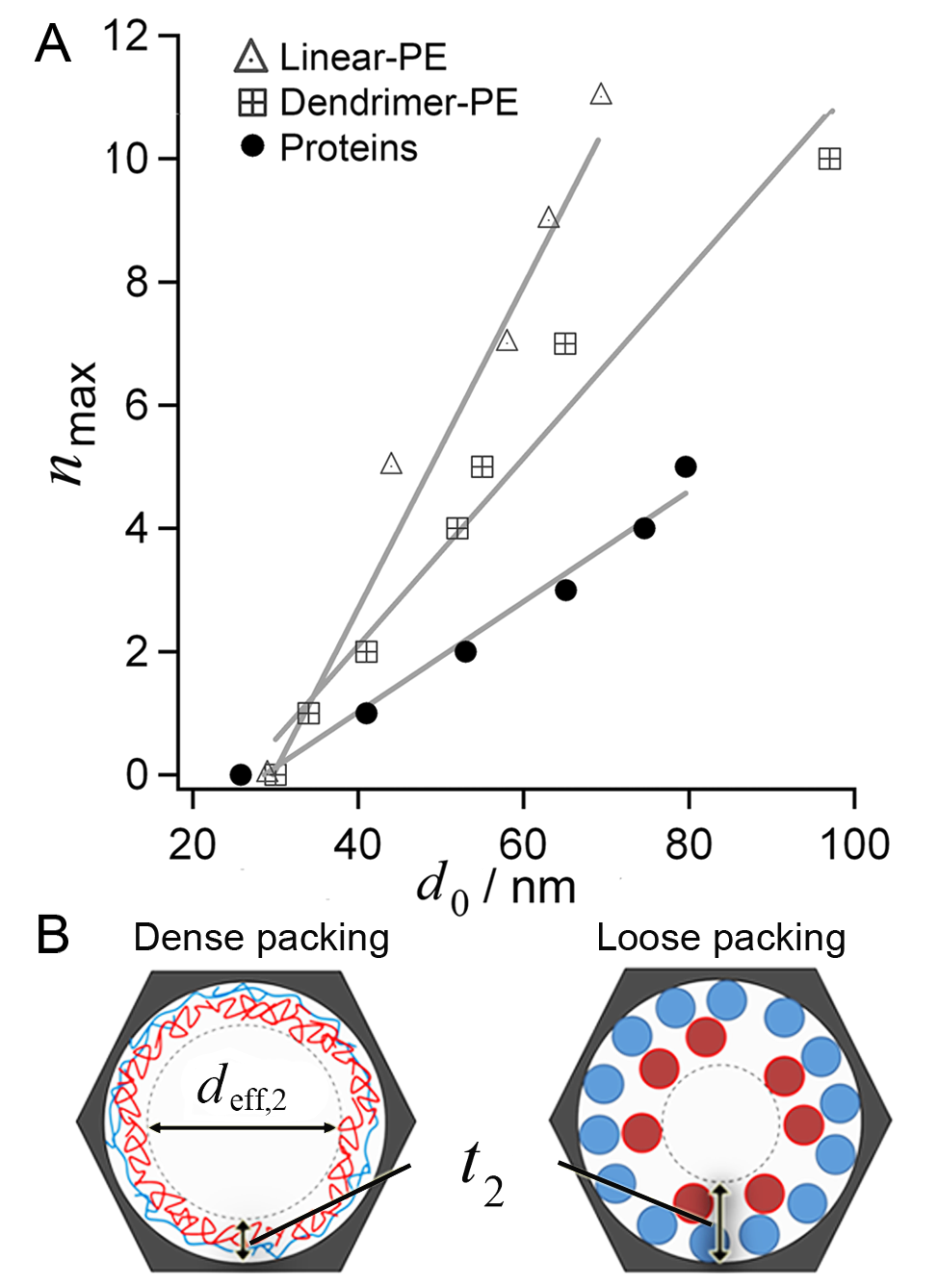
(A) Maximum number of macromolecular LbL steps (*n*_max_) for proteins, linear-PE and dendrimer-PE multilayers in AAO substrates as a function of initial *d*_0_. Lines are linear fits to the data. (B) The total volume occupied by a macromolecular layer directly influences the effective film thickness. The effective diameter for two deposited layers (*d*_eff,2_) is smaller for proteins since they form loosely packed layers. The LbL film structure determines how much volume is occupied.

In addition to the number of deposited layers, the kinetics of deposition were significantly slower for small pore diameters (*d*_0_ = 25–35 nm) for all types of macromolecules, than they were for larger pores (Supporting Information File 1, Figure S3). The transport of macromolecules within the 3–4 µm long channels was effectively inhibited on the experimental time scales studied (<60 min per deposition step) for pores with diameters of *d*_0_ = 25–35 nm. (See below for a corresponding scanning electron micrograph of these pores.) In Figure 3, *n*_max_ is plotted as a function of *d*_0_, for the globular proteins, linear-PEs and dendrimer-PEs. Linear fits to the data show that the slope, i.e., the number of maximum LbL steps, as a function of pore diameter is largest for linear-PEs, while the lowest one was achieved with proteins. The structure of the LbL films therefore influences the effective volume that each macromolecular species occupies. While the mass of the proteins and the linear-PEs is very similar, their structure, the nature of the LbL driving force, and the interaction with the AAO surface during adsorption are different. Therefore, the LbL film structure directly influences how much material can be incorporated within the nanopores. For macromolecular species that are deformable, such as the linear-PEs, compact entangled layers are typically formed, while loosely packed layers are expected for rigid, nondeformable species, such as proteins.

In all cases, the volume that the self-assembled film occupies within the pores directly influences the effective pore diameter that remains available (*d*_eff_) for macromolecular transport within the shrunken pores, after macromolecular adsorption has taken place. This consideration is illustrated in Figure 3B. The value of *d*_eff_ is expected to be larger for densely packed flexible linear molecules compared to loosely packed globular ones.

### Macromolecular interactions that limit LbL in nanopores

Each additional deposited macromolecular layer effectively shrinks the pore diameter that is available for additional macromolecules to travel within the remaining pore. As the number of LbL steps approaches saturation, i.e., *n*_max_, the effective pore diameter (*d*_eff_) reaches a certain value, upon which the cylindrical channel is insufficiently large to allow unhindered diffusion of macromolecules within the pores, a pore diameter referred to as *d*_eff,n_max_. From the experimental data, *d*_eff,n_*___*_max_ can be calculated as:

[1]
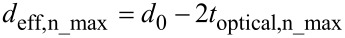


where *t*_optical,n_max_ is the cumulative optical thickness within the pores after LbL growth saturates at *n*_max_. The steric hindrance to LbL formation in cylindrical nanopores can be estimated by taking into consideration that macromolecules form adlayers that appear as large as their absolute thickness to incoming macromolecules, regardless of the surface coverage. Therefore, the value of *t*_optical,n_max_ in Equation 1 represents a measure of the film thickness that physically limits macromolecular deposition within the pores. For the linear-PEs, *d*_eff,n_max_ can be calculated according to Equation 1 to be in the range of 22–34 nm for all initial pore diameters *d*_0_. The volume occupied by the protein multilayer film, however, is underestimated by the measured cumulative *t*_optical,n_max_. This leads to overestimated *d*_eff,n_max_ values for the range of *d*_0_ tested. For all *d*_0_ values, *d*_eff,n_max_ is calculated to be larger than 40 nm. For example, considering an LbL deposition of proteins in pores with diameters of *d*_0_ = 80 nm, *t*_optical,n_max_ = 12.5 nm can be read from Figure 2A, which results in *d*_eff,n_max_ = 55 nm according to Equation 1. This cannot be correct, since three protein layers (*n*_max_ = 3) could be deposited in pores with initial pore diameters of *d*_0_ = 53 nm (Figure 2A), and therefore additional depositions would have been possible. Additional considerations are necessary to calculate a correct *d*_eff,n_max_, because loosely packed films limit the entrance of molecules to a greater extent than that estimated from the cumulative *t*_optical,n_max_. All of the species studied have similar molecular sizes in solution; however, their interactions with a surface and between the LbL layers differ significantly. Polyelectrolytes can collapse and form dense interpenetrated films, while proteins form looser aggregates due to their shape-persistent nature [39–40]. In Figure 3B, we illustrate these differences showing *d*_eff_ after the deposition of two layers of either densely or loosely packed macromolecules.

A theoretical calculation of the thickness of each individual layer *t*_calc,n_ should take into account the shape, size and nature of the macromolecular interactions with other macromolecules and with the surface. Ideally, for densely packed layers *t*_optical,n_ should equal *t*_calc,n_. For linear-PEs, *t*_calc,n_ was determined to agree with an average value of *t*_optical,n_ = 2.2 nm. However, for loosely packed assemblies *t*_optical,n_ is expected to be smaller than *t*_calc,n_. A theoretical *d*_eff,n_max_ according to:

[2]
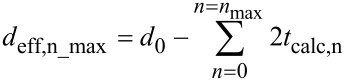


can be calculated for the loosely packed protein layers taking the protein dimensions into account. Each protein layer at the pore walls influences the incoming proteins by a reduction in the available cross-sectional area. For avidin, the individual layer thickness *t*_calc,av_ was calculated to be 5.3 nm, while for b-BSA *t*_calc,BSA_ was calculated to be 6.3 nm. These per-layer thickness values were obtained by taking the average of the three axial protein dimensions, which are 4.0 × 5.5 × 6.0 nm^3^ for avidin and 8.0 × 8.0 × 3.0 nm^3^ for BSA [41–43]. The experimentally determined protein film thicknesses with *t*_optical,av_ = 3.2 nm and *t*_optical,BSA_ = 1.1 nm are indeed considerably smaller than the theoretically calculated ones.

**Figure 4:**
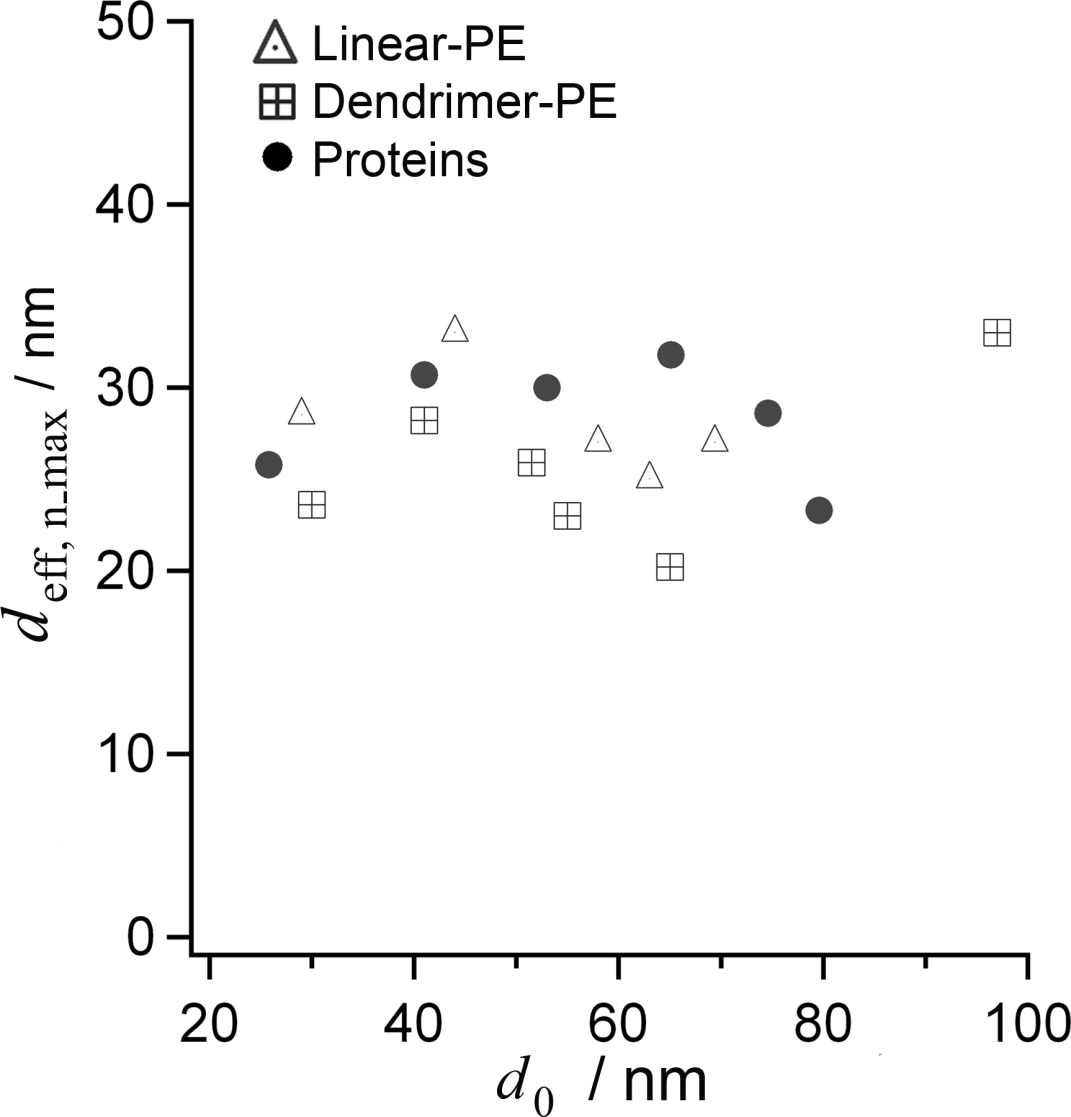
Estimated *d*_eff,n_max_ as a function of *d*_0_, for the studied linear-PEs, dendrimer-PEs and proteins. *d*_eff,n_max_ represents the reduced pore diameter when LbL growth saturates at *n*_max_. The results are in agreement with hindered deposition for a native pore diameter of *d*_0_* =* 25–30 nm.

Based on these considerations, *d*_eff,n_max_ was calculated for the different macromolecules studied as a function of the initial AAO *d*_0_ (Figure 4). For linear as well as dendrimer-polyelectrolyes, the *t*_optical,n_ values delivered a *d*_eff,n_max_ of approx. 20–35 nm (*d*_eff,nmax_ = 22–34 nm for linear PEs, and *d*_eff,nmax_ = 19–33 nm for dendrimer-PEs). For proteins, *t*_calc,n_max_ gave similar results (*d*_eff,n_max_ = 21–32 nm) for all *d*_0_ tested. Taken together, independent of the deposited layer, the minimum effective pore diameter was *d*_eff,n_max_ = 20–35 nm for the three species discussed.

**Figure 5:**
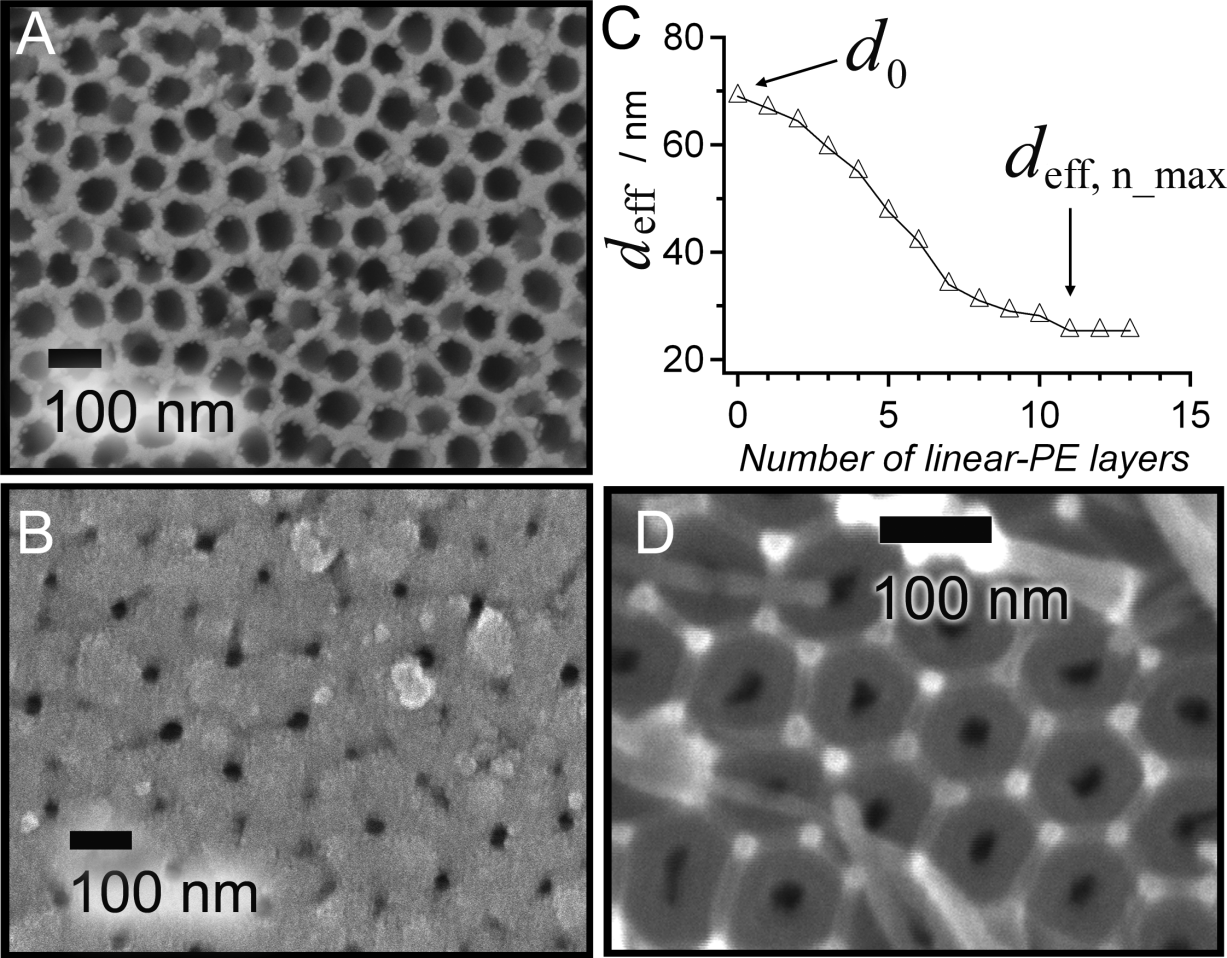
(A) SEM image of AAO with *d*_0_ = 69 nm pores before deposition of linear-PEs. (B) SEM image of AAO after 13 PSS and PAH deposition steps in AAO pores of (A). After saturation, *d*_0_ was reduced to *d*_eff,n_max_ = 24 ± 6 nm (*n* = 50). (C) *d*_eff,n_ calculated from OWS data as a function of the number of linear-PE LbL steps for the sample shown in (A) and (B). (D) SEM of AAO with a pore diameter of *d*_0_ = 25–30 nm, in which deposition was hindered (see Figure 2).

To confirm the approximated minimum effective pore diameter *d*_eff,n_max_, we recorded scanning electron microscopy (SEM) images of the substrates. Figure 5 shows SEM images of AAO nanopores with *d*_0_ = 69 nm before (A) and after the linear-PE deposition (B), at which point *d*_eff,n_max_ was reached, as shown in the corresponding OWS measurement of the reduction of *d*_eff_ depicted in Figure 5C. The initial pore diameter *d*_0_ was reduced to *d*_eff,n_max_ = 24 ± 6 nm, which matches the value obtained by the OWS experiment.

Proteins have internal, defined tertiary structures and therefore tend to deposit more loosely because molecular recognition sites that drive protein–protein interactions are not uniformly available throughout the surface. The proteins studied formed a static system, in which macromolecules effectively became locked into the configuration adopted upon initial binding between proteins, with minimal, if any, further reorganization. The flexibility of polyelectrolytes allows for chain interpenetration [44–45] and surface collapse of the polyelectrolyte structure on charged surfaces [46–48]. Furthermore, polyelectrolyte films are dynamic self-assemblies, in the sense that the internal structure of the film can undergo rearrangements to achieve optimal packing density due to flexible electrostatic interactions.

Furthermore, it is expected that the high degree of dissociation of the charged groups of the linear-PEs used in this study generates LbL multilayer films that are essentially uncharged because of the strong ionic interactions between the polyelectrolyte polymers. The film is basically precipitated onto the surface and forms compact layers. However, LbL films fabricated from weak polyelectrolytes will have a greater tendency to swell in response to ionic strength conditions, pH and solvent quality. When deposited within cylindrical nanopores, these films may exhibit a behavior intermediate between the proteins and the linear-PEs used. These differences must be considered when the optical thicknesses derived from optical measurements are used to evaluate the point at which macromolecular transport will become hindered during LbL film formation.

## Conclusions

Layer-by-layer (LbL) deposition of different macromolecular species within the cylindrical pores of anodic aluminum oxide (AAO) was strongly dependent on the size and shape of macromolecules, and on the nature of the interactions of these species with the surface and between themselves. The cylindrical pore geometry eventually becomes a physical barrier to LbL growth due to ever increasing confinement after each additional LbL step. When an effective pore diameter of 20–35 nm was reached, deposition became inhibited. This was in agreement with hindered deposition of macromolecules within native pores of diameters of *d*_0_ = 25–30 nm. AAO with different *d*_0_ were investigated to estimate the average volume that macromolecules occupy for polyelectrolytes, and proteins. The limit at which macromolecular deposition is hindered was not necessarily reflected by simply considering the optical thickness. In fact, the maximum cumulative optical thickness could only be reliably used to calculate the minimum effective pore diameter for polyelectrolytes, because they formed collapsed layers. For proteins, the multilayer LbL film thickness was approximated by using the average protein diameter as an estimate. These results showed that for the cylindrical nanopore geometry, the effective volume occupied by macromolecular species is more relevant to estimate how many LbL steps are possible before the deposition within the pore becomes hindered. In this study, we have only presented experimental results for the formation of homogeneous LbL assemblies, but the steric factors limiting the formation of heterogeneous self-assemblies can be similarly understood. Our results and experimental approach provide insight into tailoring the internal structure of multilayer LbL assemblies in nanopores towards generating multifunctional LbL films within nanoporous materials.

## Experimental

### Materials

Lyophilized avidin was purchased from Calbiochem (purity 12.9 units/mg). Biotinylated-bovine serum albumin (b-BSA) with 13 mol biotin/mol albumin, poly(sodium 4-styrenesulfonate) (PSS) (*M*_w_ = 70 kDa), poly(allylamine hydrochloride) PAH (*M*_w_ = 50–65 kDa), CuCl_2_, NaCl, and 16-mercaptohexadecanoic acid (90%) were purchased from Sigma Aldrich (St. Louis, MO, USA). (3-Aminopropyl)triethoxysilane (APTES) was purchased from Fluka (Steinheim, Germany). Oxalic acid dihydrate was from AppliChem (Darmstadt, Germany) and phosphoric acid 85% was purchased from Acros Chemicals (New Jersey, NJ, USA). Al foil (0.25 mm thick, purity: 99.999%) was purchased from Goodfellow (Huntington, UK). High refractive index LaSFN9 glass substrates (ε = 3.406 at 632.8 nm) were obtained from Hellma Optik (Halle, Germany). The UV-curable optical adhesive (NOA 83H) was purchased from Norland Products (Cranbury, NJ, USA). Ethanol was p.a. grade (VWR, France). The water used was ion exchanged and filtered by using a Millipore system (MilliQ System from Millipore, Molsheim, France; specific resistance *R* > 18 MΩ cm^−1^, pH ~5).

### AAO membranes on planar glass supports

AAO anodized from bulk aluminum foils were mounted on microscope glass slides by using an optical adhesive, according to a previously reported technique [49]. Briefly, AAO membrane thin films were fabricated by electrochemical anodization of aluminum foils after electrochemical polishing. Polished aluminum foils were anodized for 2 h in 0.3 M oxalic acid, 1 °C at 40 V. The alumina was removed with H_3_PO_4_ (5 vol %) for 2–3 h. Al foils were then anodized a second time for 1 h 35 min to obtain the desired thickness of 3.5 µm, or for 2 h to obtain 5 µm thick AAO. Al was removed by an acidic CuCl_2_ solution until the AAO became visible and no metal remained. Prior to Al removal, the AAO side was isolated from solution by immobilization onto a glass slide and sealed by using epoxy adhesive. The pore diameter of the resulting AAO membranes was widened to the desired diameter by etching in H_3_PO_4_ (5 vol %).

### Au evaporation

Au and Cr were evaporated on a Bal-Tec MCS610 evaporator equipped with a Bal-Tec QSG100 quartz film-thickness monitor. For the metal layer at the AAO bottom, 2 nm of Cr and 25 nm of Au were evaporated on the AAO barrier layer. For imaging purposes 1 nm Cr and 4 nm Au were evaporated on SEM samples.

### AAO silanization with APTES

This step was only used with the linear-PEs. For avidin, the protein was adsorbed on the unfunctionalized surface. AAO substrates were O_2_ plasma cleaned for 2 min immediately prior to gas-phase silanization to increase the surface density of OH groups. The glass slide substrates to be silanized were inserted into a glass staining jar and 50 µL of APTES were added in a glass test tube, inside the chamber. The container was covered with its glass cover and sealed, left in the oven at 130 °C for 5 min to warm, followed by 3 h under continuous vacuum.

### Surface plasmon resonance (SPR)

SPR measurements were performed on a setup operating at 632.8 nm in the Kretschmann configuration [19]. The negatively charged gold surface was obtained by immersion of an O_2_ plasma cleaned gold surface into a 10 mM mercaptohexadecanoic acid ethanolic solution for 3 h.

### Optical waveguide spectroscopy (OWS)

OWS measurements of the AAO membranes prepared on glass slides were performed on a purpose-built setup [19]. The glass-side was attached to the base of a symmetric LaSFN9 glass prism by optical immersion oil (ε = 2.89). The laser (λ = 632.8 nm) was incident through the prism-substrate assembly and reflected off the thin metal coupling layer in between the AAO and the optical adhesive as the incidence angle (θ) was varied. At specific θ values determined by the thickness and the dielectric constant of AAO (ε_AAO_), the laser was coupled into the AAO film and these waveguide modes were recorded as sharp minima in a reflectivity, *R*, versus θ scan. Transverse electric (TE) and transverse magnetic (TM) modes were indexed according to the number of nodes in their electromagnetic field distributions. ε_AAO_ and the thickness of the AAO film were obtained by fitting the angles of the waveguide mode reflectivity minima using Fresnel simulations carried out with the Winspall program [50]. Tracking the coupling angle of a mode enables real-time, in situ monitoring of changes in the dielectric constant of the film, i.e., adsorption kinetics.

The dielectric constant of AAO (ε_AAO_) that is measured by OWS includes contributions from the alumina, the pore-filling medium (e.g., buffer), and any organic thin layer coating the pore surfaces (i.e., the LbL multilayer film). The dielectric constant is related to the refractive index by ε = *n*^2^. ε_AAO_ has anisotropic components that are described by the infinite, prolate ellipsoid approximation within the Maxwell–Garnett theory, and well-described elsewhere [22,51–52]:

[3]
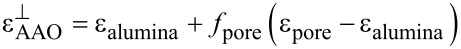


[4]
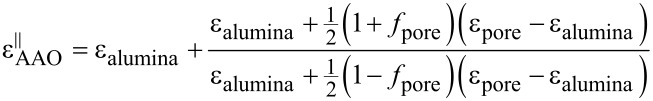



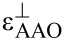
 and 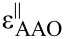 are, respectively, the dielectric constant components normal and parallel to the AAO membrane surface; *f*_pore_ is the pore volume fraction within the AAO, ε_alumina_ = 2.68 is the dielectric constant of bulk anodic alumina at λ = 632.8 nm, and ε_pore_ is the (effective) dielectric constant within the pores. For a blank AAO film in water, ε_pore_* =* ε_buffer_ = 1.78. With the addition of an organic film of proteins or linear-PEs (ε_proteins_ = 2.1, ε_linear-PEs_ = 2.15) on the internal pore surfaces, the volume within the pores is occupied by a combination of the organic material and the pore-filling buffer. Recursively applying Equation 3 and Equation 4 for the organic-filled AAO pores, using a new effective ε′_pore_ for the pore interior, provides ε_AAO_ after molecular adsorption.

### Protein and linear-PE LbL experiments

Avidin was dissolved in phosphate buffer (20 mM NaH_2_PO_4_/Na_2_HPO_4_, 100 mM NaCl, pH = 7) to obtain 0.1 mg/mL solutions. The b-BSA solutions were similarly prepared with 0.1 mg/mL concentrations. PSS and PAH solutions were prepared with 0.1 mg/mL concentrations using 500 mM NaCl in deionized water. For both macromolecules, higher ionic strengths than required were used to significantly reduce the Debye screening length and achieve optimal pore loading. The flow cell was rinsed with ethanol, followed by the buffer. The kinetics were monitored by following the change in a high-order waveguide TM-mode. The solution was passed through the flow cell (15 × 7.5 × 0.5 mm^3^) until 1.4 times the dead-volume had been washed out, and then the solution was recirculated by using a peristaltic pump. The flow rate was kept constant at 0.4 mL/min.

## Supporting Information

File 1 Additional figures.
Adsorption kinetics of dendrimer-PEs on flat and porous substrates; adsorption kinetics of avidin and PSS onto AAO with pore diameters of 25–30 nm; cumulative optical thickness of LbL dendrimer multilayer films.
