# Supplementary material for: Macromolecular shape and interactions in layer-by-layer assemblies within cylindrical nanopores
Source: Beilstein J Nanotechnol. 2012 Jun 28;3:475–84. doi: 10.3762/bjnano.3.54 (PMC3458591; doi:10.3762/bjnano.3.54)
Supplement: File 1 — Additional figures. Adsorption kinetics of dendrimer-PEs on flat and porous substrates; adsorption kinetics of avidin and PSS onto AAO with pore diameters of 25–30 nm; cumulative optical thickness of LbL dendrimer multilayer films. [file Beilstein_J_Nanotechnol-03-475-s001.pdf]

Supporting Information

for

Macromolecular shape and interactions in layer-by-layer assemblies within cylindrical nanopores

Thomas D. Lazzara[1], K. H. Aaron Lau[2], Wolfgang Knoll[3], Andreas Janshoff[4], Claudia Steinem*[1]

[1]Institute of Organic and Biomolecular Chemistry, Tammannstr. 2, 37077 Göttingen, Germany, [2]Department of Biomedical Engineering, Northwestern University, 2145 Sheridan Road, Evanston, IL 60202, USA, [3]Austrian Institute of Technology, Donau City Str. 1, 1220 Vienna, Austria, and [4]Institute of Physical Chemistry, Tammannstr. 6, 37077 Göttingen, Germany

Email: Claudia Steinem - csteine@gwdg.de

*Corresponding author

Additional figures

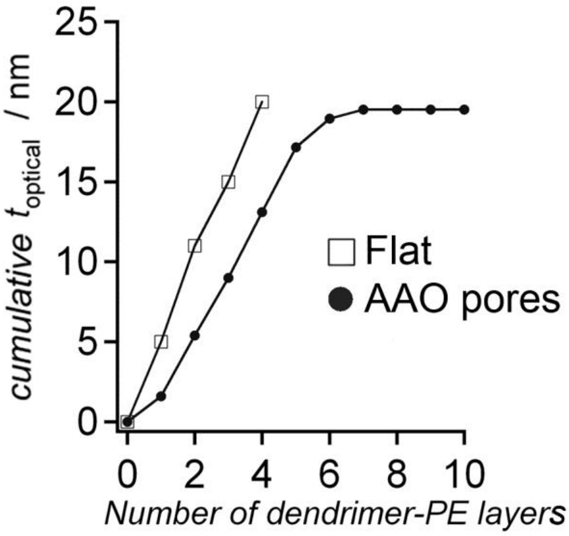

Figure S1: Adsorption kinetics of dendrimer-PEs on a flat, negatively charged, self-assembled monolayer on a gold surface, is contrasted with the adsorption on an AAO porous substrate with 65 nm diameter pores.

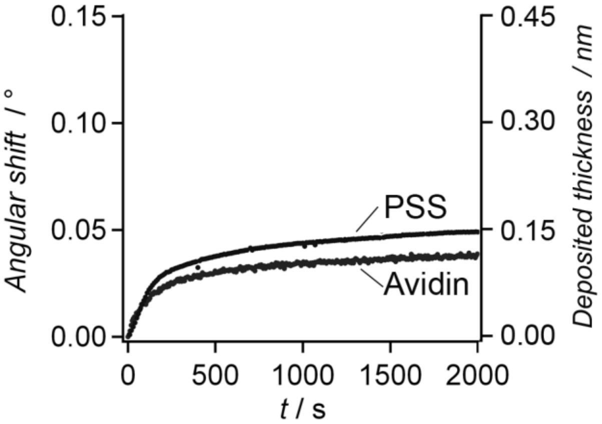

Figure S2: Adsorption kinetics of avidin and PSS onto AAO with pore diameters of about 25–30 nm. The rapid initial adsorption corresponds to deposition near the pore entrance, while the remaining slow adsorption is because of hindered transport of the macromolecules within the remainder of the pore length.

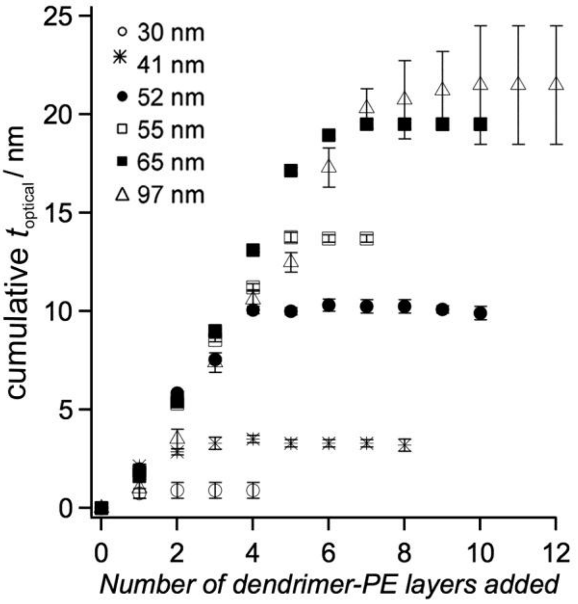

Figure S3: Cumulative optical thickness of the LbL multilayer film on the silanized inner-surface of the AAO cylindrical nanopores as a function of deposition steps and AAO pore diameter, for dendrimer-polyelectrolytes.
